# Measurement of parasitological data by quantitative real-time PCR from controlled human malaria infection trials at the Walter Reed Army Institute of Research

**DOI:** 10.1186/1475-2875-13-288

**Published:** 2014-07-28

**Authors:** Edwin Kamau, Saba Alemayehu, Karla C Feghali, Jack Komisar, Jason Regules, Jessica Cowden, Christian F Ockenhouse

**Affiliations:** 1 Military Malaria Research Program, Malaria Vaccine Branch, Walter Reed Army Institute of Research, 503 Robert Grant Ave, Silver Spring, Maryland, USA

## Abstract

**Background:**

The use of quantitative real-time PCR (qPCR) has allowed for precise quantification of parasites in the prepatent period and greatly improved the reproducibility and statistical power of controlled human malaria infection (CHMI) trials. Parasitological data presented here are from non-immunized, control-challenged subjects who participated in two CHMI trials conducted at the Walter Reed Army Institute of Research (WRAIR).

**Methods:**

Standardized sporozoite challenge was achieved through the bite of five *Anopheles stephensi* mosquitoes infected with the 3D7clone of the NF54 strain of *Plasmodium falciparum.* Blood smears were scored positive when two unambiguous parasites were found. Analysis of parasitological PCR data was performed on log-transformed data using an independent sample t-test when comparing the two studies. The multiplication rate of blood-stage parasites was estimated using the linear model.

**Results:**

On average, parasites were detected 4.91 days (95% CI = 4.190 to 5.627) before smears. The earliest parasites were detected within 120 hours (5.01 days) after challenge. Parasite densities showed consistent cyclic patterns of blood-stage parasite growth in all volunteers. The parasite multiplication rates for both studies was 8.18 (95% CI = 6.162 to 10.20). Data showed that at low parasite densities, a combination of sequestration and stochastic effects of low copy number DNA may impact qPCR detection and the parasite detection limit.

**Conclusion:**

Smear positive is an endpoint which antimalarial rescue is imperative whereas early detection of parasitological data by qPCR can allow for better anticipation of the endpoint. This would allow for early treatment to reduce clinical illness and risk for study participants. To use qPCR as the primary endpoint in CHMI trials, an algorithm of two positives by qPCR where one of the positives must have parasite density of at least 2 parasites/μL is proposed.

## Background

Controlled human malaria infection (CHMI) is increasingly being used to assess the efficacy of malaria vaccines as well as to evaluate antimalarial drug candidates [1,2]. Data obtained from CHMI are critical in the decision-making process of whether or not to proceed with more costly Phase IIb field trials [1]. CHMI trials allow for detailed evaluation of parasite growth kinetics and provide an opportunity to characterize immunological responses [1,2], which can be informative for further optimization of a vaccine or drug candidate. Studies have shown a high correlation between natural and experimental infections, which further validates the importance of using CHMI in testing new vaccines or drugs [1].

In CHMI trials, subjects are inoculated (challenged) with either *Plasmodium falciparum* or *Plasmodium vivax* sporozoites from bites of infectious, laboratory-reared, female anopheline mosquitoes. After being challenged, subjects are closely monitored for signs and symptoms of malaria such as headache, myalgia and fever. Screening for blood-stage parasites is performed by the examination of blood smears at regular intervals starting five to seven days post challenge [3]. Subjects are treated with antimalarial drugs when patent parasitaemia is confirmed by blood smears following criterion set forth in the study protocol. The detection threshold of parasites on Giemsa-stained thick films is about two to 20 parasites/μL depending on the expertise of the microscopist and the number of high-powered fields examined on a thick blood film. The comparative prepatent period of treated subjects and control subjects is used to assess efficacy of the vaccine or drug being tested [3].

The development of quantitative real-time PCR (qPCR) and other molecular techniques, which are more sensitive than microscopy has allowed for precise quantification of parasites during the prepatent period [4-6]. The qPCR data can be used to estimate liver parasite load (for pre-erythrocytic vaccines) or blood-stage multiplication rate (for erythrocytic vaccines), providing additional detailed information on the efficacy of the vaccine or drug candidate [7-10]. The mean prepatent period for control subjects challenged with sporozoites by mosquito bite is ~11 days (ranges seven to 20 days), with almost 100% of subjects bitten by five infectious mosquitoes developing patent parasitaemia [11-13]. Once the parasite emerges from the liver, the number in peripheral blood depends on multiplication rates and sequestration of parasites. Previous studies have detected parasites by PCR as early as 5.5 days after sporozoite challenge [6], with parasites detected by qPCR on average two to four days before detection by blood smears [3,6].

The use of molecular analysis has enhanced the reproducibility and statistical power of CHMI [1]. Harmonization of CHMI study methodology will further strengthen the use of molecular analysis and will allow more accurate comparison of studies conducted in different centres [3]. Further, a recent study showed that small CHMI trials even with small number of subjects are sufficiently powered to detect protective biological effects induced by pre-erythrocytic and/or blood-stage candidate vaccines if parasitaemia is measured daily by qPCR [14]. This study set-out to perform descriptive analysis of parasitological dataset from two CHMI trials recently conducted at the Walter Reed Army Institute of Research (WRAIR) in Silver Spring, MD, USA. The data presented here is from non-immunized, control-challenged volunteers only.

## Methods

### Study subjects

Samples used for analysis in this study were from infectivity control challenge subjects enrolled in two different studies conducted at WRAIR in 2012. All the subjects developed infection following challenge. These subjects did not receive any investigational product or licensed antimalarial medication prior to challenge by the bite of infectious mosquitoes. Participants were malaria-naïve adult males and females from 18–50 years of age. Protocols for both studies were approved by the WRAIR Institutional Review Board and by the Human Subjects Research and Review Board of the Surgeon General of the US Army at Fort Detrick, Maryland. Participants in both studies provided written, informed consent before screening and enrolment and had to pass an assessment of understanding.

### Infection procedures

Standardized sporozoite challenge was achieved through the bite of five *Anopheles stephensi* mosquitoes infected with the 3D7clone of the NF54 strain of *P. falciparum.* In both studies, 3D7 was prepared from the same master seed. Parasitaemia was detected by the routine daily examination of blood smears from day 5 to day 18 following challenge. Blood smears were analysed every two days from day 20 until day 28 post-challenge for any individual who had not yet become parasitaemic. Food and Drug Administration (FDA) approved oral antimalarials licensed in the USA were administered under directly observed therapy to treat uncomplicated malaria as soon as parasitaemia was detected by microscopy.

### Sample collection and processing

Samples were routinely collected every morning for blood smears and qPCR. In addition, samples were collected if a subject became symptomatic outside of scheduled, routine collection times. However, samples collected outside the routine times were not analysed in this study. Samples were collected in EDTA blood tubes and aliquoted for immediate use, or immediately stored in -20°C. For microscopic analysis, thick smears were prepared by spreading 10 μL of blood on a slide. The 10 μL of blood was smeared to make a 1 cm × 2 cm rectangle, with two such rectangles per slide. To read the smear, five passes across the 1-cm dimension of the smear were made for asymptomatic individuals and 21 passes were made for symptomatic individuals, unless the individual was found to be positive before the completion of the examination. The volume of blood examined by making five passes was 0.55 μL. Slides were examined by two independent readers under oil immersion at 1000× magnification. Blood smears were scored positive when two unambiguous parasites were found. The same team of microscopists was responsible for smear analysis in both studies.

### Real-time PCR assay

The qPCR analysis was done in real-time using reagents, procedures and methods as previously described [15,16]. The assay characteristics, including the limit of detection, have been described previously [17]. Samples were analysed using the genus-specific PLU assay and endogenous control RNaseP assay, which was performed as a multiplex assay. The WHO International Standard for *P. falciparum* DNA (obtained from National Institute for Biological Standards and Control; NIBSC, Hertfordshire, UK. Referred to herein as NIBSC standard), plasmid DNA or combination of both was used as standard for quantification of the parasite density in all the qPCR assays performed. Details of the reagents and analysis methods have been described previously [15,16]. Briefly, DNA was purified from 200 μL whole blood and eluted in 200 μL elution buffer or water using EZ1 automated purification system (Qiagen, Valencia, CA, USA) with the EZ1 DNA blood kit (Qiagen) following the manufacturer’s recommendation. For qPCR assay, 1 μL DNA was added in 4 μL master mix. Each assay was run on a 96-well plate with NIBSC standard. The performance of the NIBSC standard and RNaseP for individual assays was assessed to determine the success of the analysis. The amplification plot for each assay was individually assessed to ensure true amplification of the assay when C_q_ (quantification cycle, can also be referred to as the threshold cycle [C_t_]) values were obtained. The qPCR assays were performed within four hours of blood collection.

### Data analysis

Statistical analyses were performed using Graph-Pad Prism (San Diego, CA, USA) and STATA software version 10 (College Station, TX, USA). Prepatent period was defined as length of time between challenge and detection of a positive blood smear. Analysis of parasitological PCR data was performed on log-transformed data using an independent sample t-test when comparing the two studies. The multiplication rate of blood-stage parasites was estimated using the linear model as recently described [18]. The cycles of parasite growth were estimated as follows: the first cycle started the first day parasites were detected until day 8, the second cycle was day 9–10, the third cycle was day 11–12, and the fourth cycle was day 13–14 where applicable.

## Results

### Time to parasitaemia

**Figure 1:**
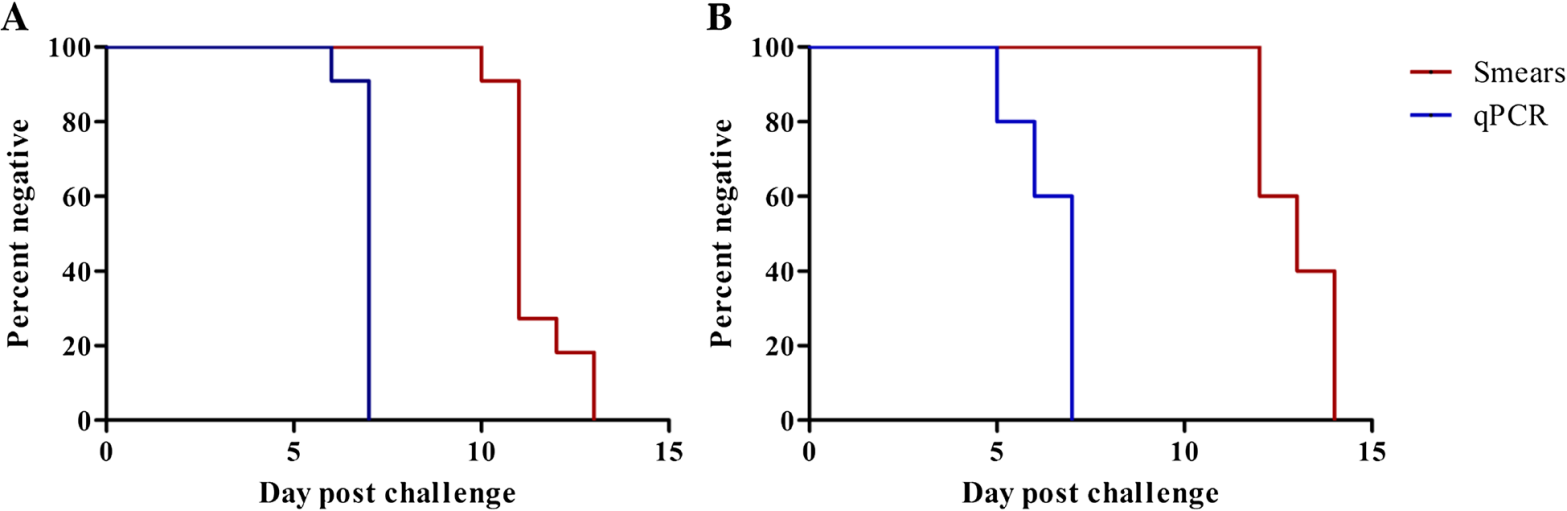
**Time to smear and qPCR positive.** Survival curves showing the percent of subjects negative by both smears and qPCR. **(A)** first study and **(B)** second study.

Data from 16 unvaccinated infectivity control subjects enrolled in two different malaria vaccine trials at the WRAIR were analysed by qPCR. Table 1 shows the mean number of days before detection of parasites in both studies by blood smears and qPCR. The earliest parasites detected were in a subject from the second study where parasites were detected by qPCR 120 hours (5.01 days) after the time of challenge, with a parasite density of 0.174 parasite/μL. Parasites were detected by qPCR in all study subjects within seven days following challenge. In the first study, parasites were detected on average 4.34 days (95% CI = 3.70 to 4.98) before smears, whereas in the second study, parasites were detected on average 6.17 (95% CI = 4.57 to 7.77) days before smears. Figure 1A and B are survival curves showing the per cent of subjects negative by both smears and qPCR for first and second study.

**Table 1:** Time to parasitaemia (days), showing the number of subjects from the first and second study

	**Mean**	**Std Dev**	**Max**	**Min**	**Median**	**25%**	**75%**	**P Value**
**First study (n = 11)**								
**Smear**	11.34	0.93	13.02	10.11	10.96	10.91	11.97	0.0001
**qPCR**	7.01	0.07	7.12	6.91	6.98	6.95	7.07	
**Second study (n = 5)**								
**Smear**	12.54	1.33	14.00	10.96	11.98	11.43	13.94	0.0004
**qPCR**	6.38	0.89	7.01	5.01	6.93	5.47	7.00	

### Parasite density

**Figure 2:**
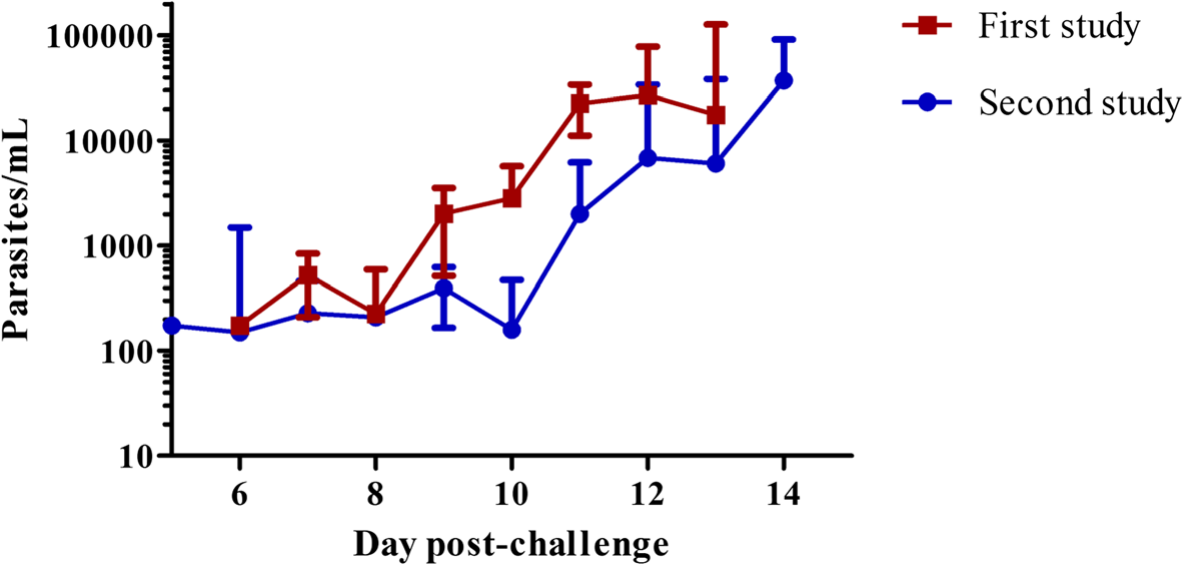
**Parasite density as measured by qPCR.** The geometric mean is shown from both studies with 95% confidence intervals, from the day which qPCR was initiated (day 5 after challenge) until the day the subjects became smear positive and treatment was initiated.

Parasite densities showed consistent cyclic patterns of blood-stage parasite growth in all volunteers from both studies. Figure 2 shows the geometric mean of parasite density from both studies. Parasites in the second study were detected sooner by qPCR compared to the first study and at a lower density. Parasite geometric mean density first cycle was 0.444 parasites/μL (95% CI = 0.274 to 0.718) for the first study and 0.202 parasites/μL (95% CI = 0.107 to 0.383) for the second study. Geometric mean parasite density by smears for the first study was 3.29 parasites/μL (95% CI 2.00 to 5.42) and 1.68 (95% CI = 0.97 to 2.92) for the second study. Geometric mean parasite density by qPCR analysis on the first day of qPCR positivity for the first study was 0.394 parasites/μL (95% CI = 0.272 to 0.572) and 0.174 parasites/μL (95% CI = 0.079 to 0.383) for second study. Interestingly, the geometric mean parasite density by qPCR at the day of smear positive for first study was 23.92 parasites/μL (95% CI = 16.43 to 34.80) and 35.74 parasites/μL (95% CI = 25.43 to 50.23) for second study.

### Parasite multiplication rate and growth

**Figure 3:**
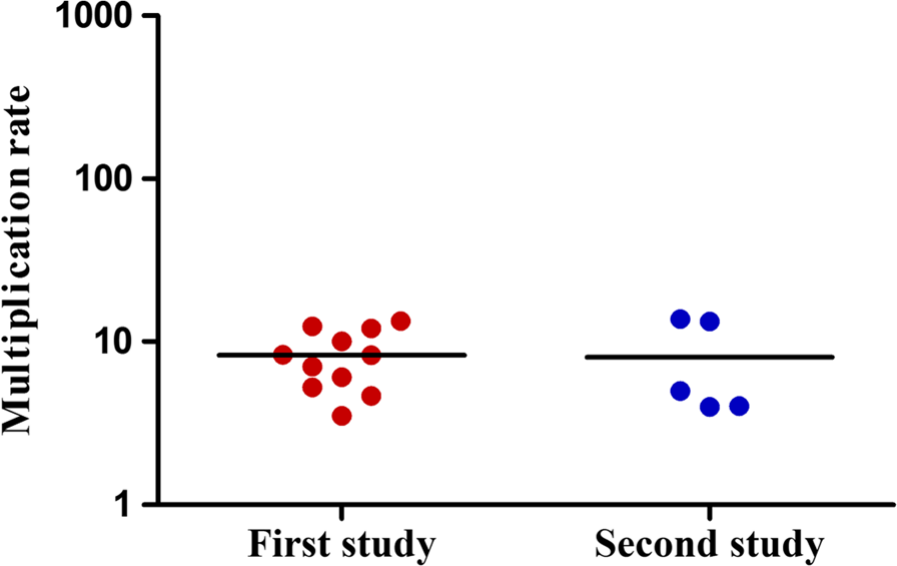
**Parasite multiplication rate for trials.** Individual data are plotted, lines represent mean.

**Figure 4:**
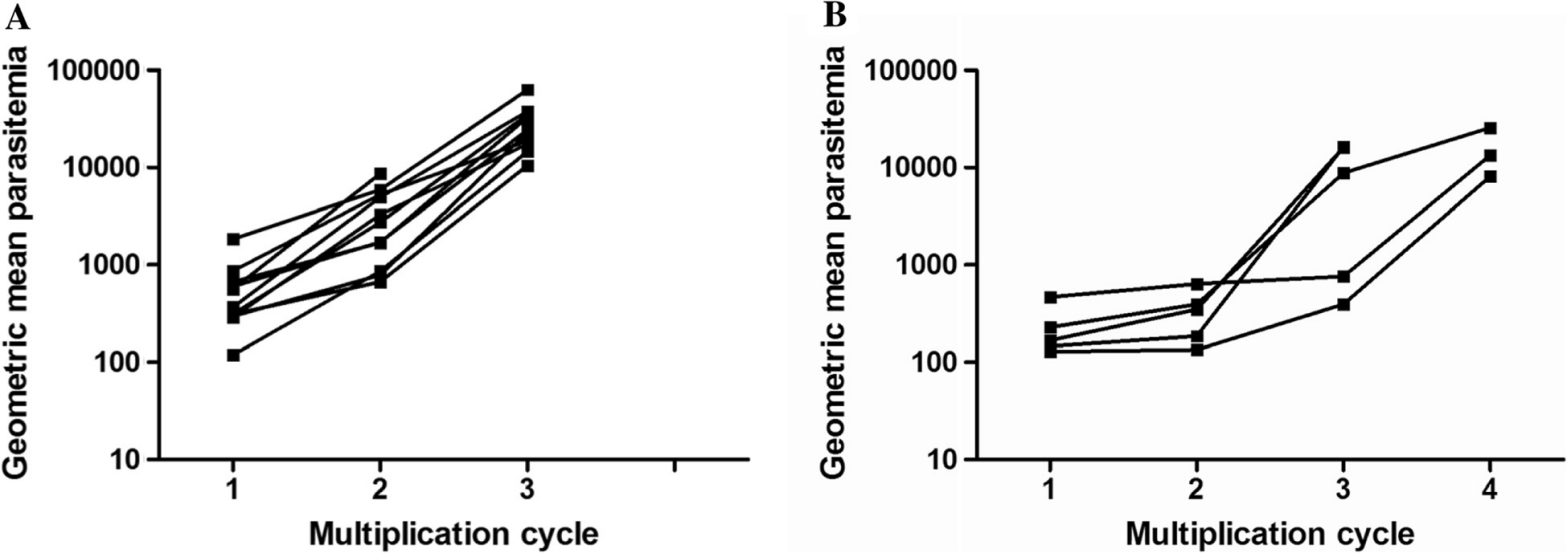
**Geometric mean parasite density per multiplication cycle for individual volunteers.** Panel **A** shows data from the first study and panel **B** shows data from the second study.

Figure 3 shows parasite multiplication rate (PMR) for the first and second study. The mean PMR for first and second studies were 8.26 (range = 3.5-13.37) and 8.00 (range = 3.98-13.77), respectively. However, parasites in the second study, which were detected sooner and at a lower parasite density, remained low until day 11 (Figure 2). The geometric mean parasite density on day 11 was 22.57 parasites/μL (CI = 15.37-33.15) for the first study compared to 2.02 parasites/μL (CI = 0.37-11.03) for second study. This is reflected by the fact that on day 10 and 11, eight out of 11 (seven on day 11) subjects were found parasite positive by smear for the first study whereas for the second study, subjects did not become smear positive until day 12 (two subjects), day 13 (one subject) and day 14 (two subjects). Table 2 shows the geometric mean parasite density in parasites/μL in the first, second, third, and fourth cycles for first and second study. Figure 4A and B show the geometric mean parasite density in parasites/mL per multiplication cycle for individual subjects. Data clearly indicate that growth rates for the second study were initially slow in the first and second or third cycle but increased rapidly in the third or fourth cycle.

**Table 2:** Parasite cycles (parasite/μL)

	**1st cycle (95% CI)**	**2nd cycle (95% CI)**	**3rd cycle (95% CI)**	**4th cycle (95% CI)**
**First study**	0.44 (0.27 to 0.72)	2.41 (1.33 to 4.37)	24.55 (16.87 to 35.73)	Nil
**Second study**	0.20 (0.11 to 0.38)	0.29 (0.14 to 0.64)	3.71 (0.41 to 33.86)	14.15 (3.36 to 59.66)

### Effect of parasite sequestration in qPCR detection

The numbers of parasites in peripheral blood depend on multiplication rates and sequestration. At low parasite density, stochastic or random sampling effects of low copy number of DNA during PCR amplification leads to fluctuation of results between replicate analyses. A combination of sequestration and stochastic effects may impact qPCR detection and the C_q_ values obtained at low densities. Table 3 shows C_q_ values from two subjects for whom qPCR analysis was repeated in eight replicates for two consecutive days. The initial qPCR runs were done in triplicate (shown in bold). The qPCR assay for Subject ID 01 performed on 02FEB, demonstrates stochastic effects due to low parasite density. When the qPCR assay was initially run in triplicate on 02FEB, only one C_q_ value out of the three replicates was detected at cycle 42.08, which is outside the set assay limit of C_q_ value of 40, and therefore it was considered there was no amplification. However, when repeated in eight replicates, five C_q_ values were recorded with standard deviation (SD) of 0.983. As the parasite multiplied and density increased (24 hours), all C_q_ values were within the detectable range (with lower SD) the following day. When initially run in triplicate, subject ID 02 had one C_q_ value out of the three replicates detected on 02FEB at cycle 34.47 but none was detected on 03FEB. However, when repeated in eight replicates, five C_q_ values were recorded on 02FEB and four C_q_ values were recorded on 03FEB. These data clearly demonstrate that a negative C_q_ value can be due to a lack of parasites in the sample, low parasite density because it is early in the parasite life cycle, low numbers of parasites due to sequestration, or the stochastic effects of low copy number DNA template, which demonstrates the limits of qPCR (molecular) assays.

**Table 3:** Effect of parasite sequestration in qPCR detection

**ID**	**Date**	**C**_**q**_	**C**_**q **_**Mean**	**C**_**q **_**SD**	**ID**	**Date**	**C**_**q**_	**C**_**q **_**Mean**	**C**_**q **_**SD**
**01 02FEB**		**42.08**	**42.08**		**01 03FEB**		**30.58**	**31.02**	**0.49**
		**Und**	**42.08**				**31.54**	**31.02**	**0.49**
		**Und**	**42.08**				**30.92**	**31.02**	**0.49**
		Und	33.76	0.98			31.05	30.86	0.32
		32.97	33.76	0.98			30.51	30.86	0.32
		34.92	33.76	0.98			31.24	30.86	0.32
		Und	33.76	0.98			30.95	30.86	0.32
		34.74	33.76	0.98			30.30	30.86	0.32
		33.00	33.76	0.98			31.18	30.86	0.32
		33.13	33.76	0.98			30.82	30.86	0.32
		Und	33.76	0.98			30.82	30.86	0.32
**ID**	**Date**	**C**_**q**_	**C**_**q **_**Mean**	**C**_**q **_**SD**	**ID**	**Date**	**C**_**q**_	**C**_**q **_**Mean**	**C**_**q **_**SD**
**02 02FEB**		**34.47**	**34.47**		**02 03FEB**		**Und**		
		**Und**	**34.47**				**Und**		
		**Und**	**34.47**				**Und**		
		37.30	36.22	2.01			33.93	34.34	0.68
		Und	36.22	2.01			34.99	34.34	0.68
		33.28	36.22	2.01			Und	34.34	0.68
		37.30	36.22	2.01			Und	34.34	0.68
		35.05	36.22	2.01			Und	34.34	0.68
		Und	36.22	2.01			34.84	34.34	0.68
		Und	36.22	2.01			33.60	34.34	0.68
		38.17	36.22	2.01			Und	34.34	0.68
							

Bold C_q_ values indicate the initial qPCR runs which were done in triplicate.

## Discussion

This study show detailed estimations of critical parameters in the parasite life cycle from CHMI studies conducted at WRAIR using qPCR measurements of parasitaemia in infectivity control (non-vaccinated), malaria-naive (non-immune) volunteers. Parasite analysis was done in real time and results were available within four to six hours from the time of blood collection. Parasites were detected in all volunteers who eventually developed malaria within seven days of the challenge, with the earliest detection being at 5.01 days. Previous studies have demonstrated parasites are detected by qPCR on average of two to four days before microscopy [3,6]. Interestingly, parasites were detected on average 6.17 days before microscopy in the second study. In one of the subjects, parasites were detected 7.93 days before detection by microscopy. This is the largest interval that has been reported in CHMI between qPCR positivity and blood smear parasite detection. In the first study, parasites were detected on average 4.34 days before microscopy. In both studies, there were no false qPCR positives based on data obtained from infectivity controls and vaccine recipients (data to be reported elsewhere).

In CHMI studies, the primary endpoint of blood-stage infection is the first detection of parasites by microscopy. Since CHMI studies mostly recruit malaria-naïve, healthy volunteers, there has been increasing discussion about whether qPCR should replace microscopy as the primary endpoint for clinical diagnosis in order to reduce malaria symptomatology, clinical illness and risk for study participants. Use of qPCR for endpoint diagnosis with the first appearance of PCR positive sample may be suitable for pre-erythrocytic (liver stage) vaccines. However, it would be less useful in the evaluation of vaccines and drugs with blood-stage activity as efficacy for these can only be obtained by evaluating blood-stage parasite growth over a sufficient period of time [14]. Treatment based on qPCR would decrease the time window required for detailed analysis and estimation of critical parameters in the parasite life cycle required for measuring the efficacy of blood-stage vaccines [3]. Development of a qPCR treatment algorithm is therefore critical in keeping the time window long enough to evaluate vaccine efficacy yet short enough to treat volunteers in time (before they would be smear positive) to reduce clinical illness. In this study, the geometric mean parasite density by qPCR on the day of first positive blood smear was 23.92 parasites/μL (95% CI = 16.43-34.80) and 35.74 parasites/μL (95% CI = 25.43-50.23) for the first and second study, respectively. The geometric mean parasite density by qPCR on the day prior to the first positive smear was 5.78 parasites/μL (95% CI = 2.92-11.43) and 6.12 parasites/μL (95% CI = 1.87-20.00) for first and second study, respectively. In a study by Roestenberg *et al.* which compared parasitological data from CHMI studies, the lowest geometric mean peak parasitaemia detected was 7.08 parasites/μL [10]. In another study, using pooled data, the geometric mean parasitaemia and second and third cycles were determined to be 4.49 and 11.37 parasites/μL, respectively [14]. Most molecular assays used in CHMI studies have a threshold of about 0.02 parasites/μL [4,6,17,19]. To use qPCR for end-point diagnosis in CHMI, an algorithm of two positives, not necessarily on consecutive days (because of parasite sequestration), where one of the positives must have parasite density of at minimum two parasites/μL is proposed. This is a parasite density that is well within range as detected by qPCR at the day of positive smears in this and other studies. This will allow enough time window for analysis of the effect of the vaccine while still allowing initiation of antimalarial treatment one to two days before becoming smear positive. For this algorithm to be effective however, it is important that CHMI centres use harmonized qPCR analysis and common reference standard DNA for determination of parasite density. In addition, as more data are collected from more centres, the recommendations regarding minimum parasite density should be adjusted.

Inter-individual variation is probably the most important factor in determining the sensitivity of sporozoite challenge trials, which might be influenced by the parasite inoculum size, parasite fitness, and human (innate) immune factors [14]. Use of malaria**-**naïve volunteers in CHMI studies helps reduce variance due to immune factors. However, parasite inoculum size and parasite fitness is still an issue in sporozoite challenge trials. The variability in prepatent period, parasite growth rates and densities found between first and second study could be due to the infectivity of the sporozoites, the number of sporozoites per mosquito, or the number of sporozoites inoculated by the bites of five infected mosquitoes. This has been shown to vary from centre to centre, between trials at the same centre, and even between individuals in the same trial at the same centre [20]. Although it has been suggested that the use of vialed sporozoites administered by needle and syringe for challenge could reduce these variations [3,20], it is important to be cautious since this is not a natural route of sporozoite infection and will undoubtedly have immune response implications [1]. In addition, it will not address the issues of variation in parasite fitness and innate host variability.

## Conclusion

This study demonstrates reproducibility of CHMI studies conducted at the WRAIR and the use of qPCR for measurements of parasitaemia. An algorithm that can be used for qPCR as an endpoint diagnosis has been proposed. Use of qPCR as an endpoint diagnosis is likely to appeal to FDA and other authorities because it will reduce malaria symptomatology, clinical illness and risk for study participants. However, all the benefits of using qPCR as an endpoint diagnosis must be weighed against the legacy of using smears as the endpoint and the backward compatibility with data collected from previous studies.

## Competing interests

The authors declare that they have no competing interests.

## Authors’ contributions

EK and CFO conceived the project idea. EK, SA KCF designed and executed the experiments. JK was responsible for microscopy work. JR and JC were the principal investigators of the clinical trials. EK wrote the manuscript. SA, KCF, JK, JR and JC reviewed the manuscript. All authors approved the final version of the manuscript.
